# In vitro and in vivo properties of CD133 expressing cells from human lung cancer cell lines

**DOI:** 10.1186/2162-3619-2-16

**Published:** 2013-06-06

**Authors:** Ping Wang, Zhenhe Suo, Mengyu Wang, Hanne K Høifødt, Øystein Fodstad, Gustav Gaudernack, Gunnar Kvalheim

**Affiliations:** 1 Department of Cellular Therapy, Oslo University Hospital, Radiumhospitalet, Oslo, Norway; 2 Department of Immunology, Institute of Cancer Research, Oslo University Hospital, Radiumhospitalet, Oslo, Norway; 3 Department of Pathology, Oslo University Hospital, Radiumhospitalet, Oslo, Norway; 4 Department of Tumor Biology, Institute of Cancer Research, Oslo University Hospital, Radiumhospitalet, Oslo, Norway; 5 Faculty of Medicine, University of Oslo, Oslo, Norway; 6 Department of Hematology, Henan Tumor Hospital, Zhengzhou, P. R. China

**Keywords:** Cancer stem cells, Lung cancer cell lines, CD133, CD44, Side population

## Abstract

**Background:**

Tumor development is recently hypothesized to depend on a rare cell population with stem cell properties, such cells are called cancer stem cells (CSCs) or tumor-initiating cells (TICs). From various cancer tissues or cancer cell lines, CD133 expressing cells were found to define a unique CSC/TIC phenotype. To study whether that also could be the case in lung cancer, we examined different lung cancer cell lines for CD133 expression.

**Results:**

Among the 4 cell lines studied, only the cell line LC-42 expressed CD133. Therefore, LC-42 was further characterized and studied with special emphasis on identifying the presence of CD133^+^ CSCs/TICs. FACS sorted CD133^high^ and CD133^dim^ subpopulations from LC-42 showed no differences in soft agar colony-forming capacity and spheres-forming capacity in serum-free cultures. LC-42 cells contained Side Population (SP), and only SP cells were able to form spheres. Furthermore, Nanog expression was significantly higher in SP than in non-SP. However, no difference was observed of CD133 expression in SP and non-SP. When CD133^high^ and CD133^dim^ cells were serially xeno-transplanted in NOD/SCID mice, both formed tumours similar to their parental LC-42 cells. There were no expression differences for NANOG, OCT4 and SOX2 examined immunohistochemically in the xenografts from both cell fractions.

**Conclusion:**

Our data do not show a difference in tumorigenic potential of CD133^high^ and CD133^dim^ cells with respect to any of the parameters analyzed *in vitro* and *in viv*o, suggesting that CD133 expression is not restricted to cancer-initiating cells in the human lung cancer cell line LC-42.

## Introduction

Lung cancer is the number one cause of cancer-related deaths by far in the western countries, even more frequently seen than breast, prostate, and colon cancer combined. Late appearance of clinical symptoms from a lung cancer is the main reason for that the majority of the patients have advanced disease at diagnoses. In Norway, only 7% of the lung cancers are candidate for radical surgery. New chemotherapy combinations and improved radiotherapy methods have not improved the prognosis of the lung cancer patients and the 5 year events-free survival is still <15% [1].

Traditional cancer treatments against lung cancer have been developed based on targeting proliferating tumor cells, but this therapy is proven ineffective since recurrence of the disease occurs often when the therapy is stopped. Tumor growth is recently hypothesized to depend on a rare cell population within a tumor propagating in vivo, and these cells are responsible for radio/chemotherapy resistance and are the cause of recurrence and metastasis after therapy. These cells, denominated cancer stem cells (CSCs) or tumor-initiating cells (TICs), is believed to share similar functions as its normal stem cell counterparts [2,3], and can therefore be defined by their self- renewal capacity and ability to differentiate. Furthermore, they can be identified by stem cell associated antigen expression and be sorted with a fluorescence-activated cell sorter (FACS) for further molecular and biological studies. CSCs/TICs were first identified in human leukemia using different cell surface markers and cell sorting. A subpopulation of acute myeloid leukemia cells expressing the antigens CD34^+^CD38^-^ assembled characteristics and properties as stem cell, both in vitro and in immunodeficient mice [4,5]. Since then, several groups have identified other surface markers being associated with CSC/TIC in solid tumors like breast cancer [6], brain tumor [7], melanoma [8], colon cancer [9,10], pancreatic cancer [11,12], prostate cancer [13] and head and neck squamous cell carcinoma [14].

Normal lung tissue is composed of different types of cells, basal mucous secretary cells in the trachea and bronchi, Clara cells in the bronchioles and type 1 and type 2 pneumocytes in the alveoli. These mature cells derive from the differentiation of lineage-restricted lung progenitor cells, which originate from undifferentiated multipotent lung stem cells. Multipotent lung stem cells have been identified throughout the airways and are thought to be responsible for local tissue maintenance and tissue repair. In 2005, Kim CF *et al.* became the first one to identify bronchialveolar stem cells (BASCs) localized at the bronchioalveolar duct junction. These putative stem cells formed the bronchiolar Clara cells and alveolar cells of the distal lung. Interestingly, the same investigators also found, in a mouse model, that tumor initiating cells of lung adenocarcinoma originated from malignant BASCs [15]. Human multipotent lung stem cells and CSCs that give rise to all subtypes of lung cancers remain to be finally defined.

CD133 is a surface protein with five transmembrane domains and was initially recognized as an antigen expressed on human stem and progenitor haematopoietic cells [16]. Soon after that, the antibody CD133 was used to isolate endothelial progenitor cells [17] and central nervous system stem cells [18]. Low numbers of CD133^+^ cells were found in most adult human organs using the original CD133 antibody (AC133) suggesting that this antigen can be defined as an organ specific stem cell marker. To date, CD133 antigen expression itself or combined with other markers has been used to identify and isolate tumor cells with stem cell characteristics in brain tumor [7], colon cancer [9,10], prostate cancer [13], pancreatic cancer [11], melanoma [19] and lung cancer [20]. When sorted CD133^+^ tumor cells from these types of tumors were tested in immunodeficient mice, they showed unique ability to propagate tumors.

Despite the fact that CD133 antigen expression can be used to identify and purify CSC population in some types of solid tumors, recently, several groups have challenged the idea that CD133 antigen expression can be used as a general tumor stem cell marker. In colon cancer, CSCs are not uniquely CD133^+^ since CD133^-^ cells of the tumors were able to produce CD133^+^ cells as well [21,22].

In this paper, we have used 4 different human lung cancer cell lines to study subpopulations of cells with CSC characteristics. Among the 4 human lung cancer cell lines, the LC-42, which was established from a lymph node metastasis of a squamous cell lung cancer, expressed the CD133 antigen while the other cell lines were only weakly or negative for CD133 expression. To investigate the properties of LC-42 cells in relation to the CD133 antigen expression, different in vitro and in vivo studies were performed. In contrast to previous reports, we found that sorted CD133^high^ and CD133^dim^ LC-42 cells had equal stemness gene expression profile, colony and sphere formation capabilities in vitro and equal tumorigenic growth pattern in NOD/SCID mice.

## Results

### Expression of stem cell related markers in the lung cancer cell lines LC-42, HTB-182, EKVX and SELS

**Figure 1:**
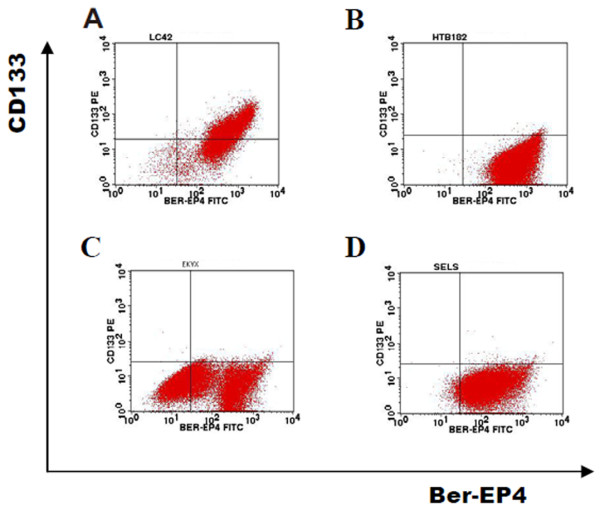
**FACS analysis of CD133 antigen expression in the 4 different lung cancer cell lines.** One representative experiment of the FACS analysis of CD133 antigen expression in the 4 different lung cancer cell lines: LC-42, HTB-182, EKVX and SELS. CD133 antigen expression was found to be strong in the cell line LC-42 (56.89±6.26%, n= 5), while were weakly expressed on the remaining cell lines and were similar to the isotype control. The pan-epithelia marker Ber-EP4 was widely homogenous expressed in all the cell lines except EKVX.

Based on the current knowledge on putative stem cell markers, the cell line LC-42 and other three lung cancer cell lines were phenotyped by flow cytometry and IHC. In flowcytometry analysis, CD34 and CD45 were expressed extremely low in these lung cancer cell lines (data not shown), while the chemokine receptor CXCR4 was highly expressed in the HTB-182 cell line, but not expressed in the remaining cell lines. The CD117, CD31 and VEGFR were weakly or negatively expressed in all lung cancer cell lines. The epithelium specific marker Ber-EP4 was expressed heterogeneously in all the cell lines (Figure 1).

**Figure 2:**
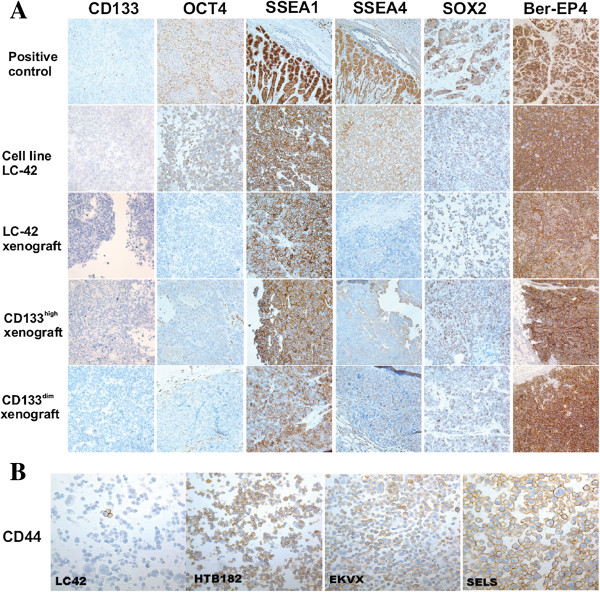
**IHC analysis of CD133, OCT4, SSEA1, SSEA4 and SOX2 in xenografts from different cell fractions. A.** IHC analysis of CD133, and the stem cell related transcription factors OCT4, SSEA1, SSEA4 and SOX2 in parental LC-42 cells, corresponding xenograft, and xenografts derived from CD133^high^ and CD133^dim^ cells. The xenografts resemble their parental LC-42 cells for the expressions of CD133, OCT4, SSEA1, SSEA4 and SOX2. **B.** IHC analysis of CD44 expression in 4 lung cancer cell lines LC-42, HTB-182, EKVX and SELS. All the representative images are at 40× magnifications.

Interestingly, CD133, which has been suggested to be a marker of tumor stem cells of different types of tumors, was highly expressed (56.89±6.26%) in the LC-42 lung cancer cells, while the EKVX, SELS and HTB-182 cells showed a weak expression of the CD133 antigen (0.39±0.30%, 0.43± 0.17% and 1.07±0.57%, respectively) (Table 1). In contrast, the CD44 protein was strongly expressed in the EKVX, SELS and HTB-182 cells (97.00±1.27%, 99.8±0.09% and 99.56±0.34%, respectively) while only 3.75±0.08% of the LC-42 cells were CD44 positive (Table 1). The expression of CD133 and CD44 in the 4 cell lines were further verified by IHC. The IHC analysis of CD133 expression using the clone AC133 (Figure 2A) showed consistent positive results with the analysis done on FACS in the LC-42 cells, while the other 3 lung cancer cell lines EKVX, SELS and HTB-182 all had completely negative immunoreactivity for the CD133 antibody. The expression of the antigen CD44 was only positive in some single cells in the LC-42 cell line while it was broadly expressed in the lung cancer cell lines EKVX, SELS and HTB-182 (Figure 2B).

**Table 1:** FACS analysis of the stem cell related markers in the 4 lung cancer cell lines

**Cell line marker**	**LC-42**	**HTB-182**	**EKVX**	**SELS**
**CD133**	56.89±6.26	1.07±0.57	0.39±0.30	0.43± 0.17
**CD44**	3.75±0.08	99.56±0.34	97.00±1.27	99.8±0.09
**CXCR4**	0.44±0.37	13.10±6.42	0.13±0.03	0.33±0.12
**Ber-EP4**	99,03±0,61	99,74±0,21	48.22±2.70	92,87±0,43
**CD117**	1.47±0.72	0.47±0.35	0.49±0.16	0.13±0.05
**CD31**	0.50±0.08	0.74±0.72	0.53±0.25	0.12±0.03
**VEGF R2**	0.48±0.14	0.89±0.76	0.13±0.03	0.20±0.08

The values (mean±SD, n≥3) of stem cell markers on each lung cancer cell line represent the average of at least three individual flow cytometry analyses.

Expression of stem cell related transcription factors OCT4, SOX2, SSEA1, and SSEA4 were analyzed on sections from the LC-42 and the other three lung cancer cell lines. The staining of each one showed various immunoreactivity among the 4 lung cancer cell lines (Additional file 1: Table S1). However, for the cell line LC-42, all these transcriptfactors were found moderate or strong positive expressed. The pan-epithelial marker Ber-EP4 was widely expressed in all the 4 cell lines (Figure 2A) (Table 2).

**Table 2:** **The summary of the IHC analysis in Figure **2

**Marker material**	**CD133**	**OCT4**	**SSEA1**	**SSEA4**	**SOX2**	**Ber-EP4**
**LC-42 cell line**	+	+	+	+	+	+
**LC-42 cells xenograft**	+	+	+	+	+	+
**CD133**^**high **^**cells xenograft**	+	+	+	+	+	+
**CD133**^**dim**^**cells xenograft**	+	+	+	+	+	+

IHC analysis of CD133, and stem cell related transcription factors OCT4, SSEA1, SSEA4 and SOX2 in parental LC-42 cells, corresponding xenograft, and xenografts derived from CD133^high^ and CD133^dim^ cells.

### Colony and sphere formation of CD133 sorted LC-42 cells

**Figure 3:**
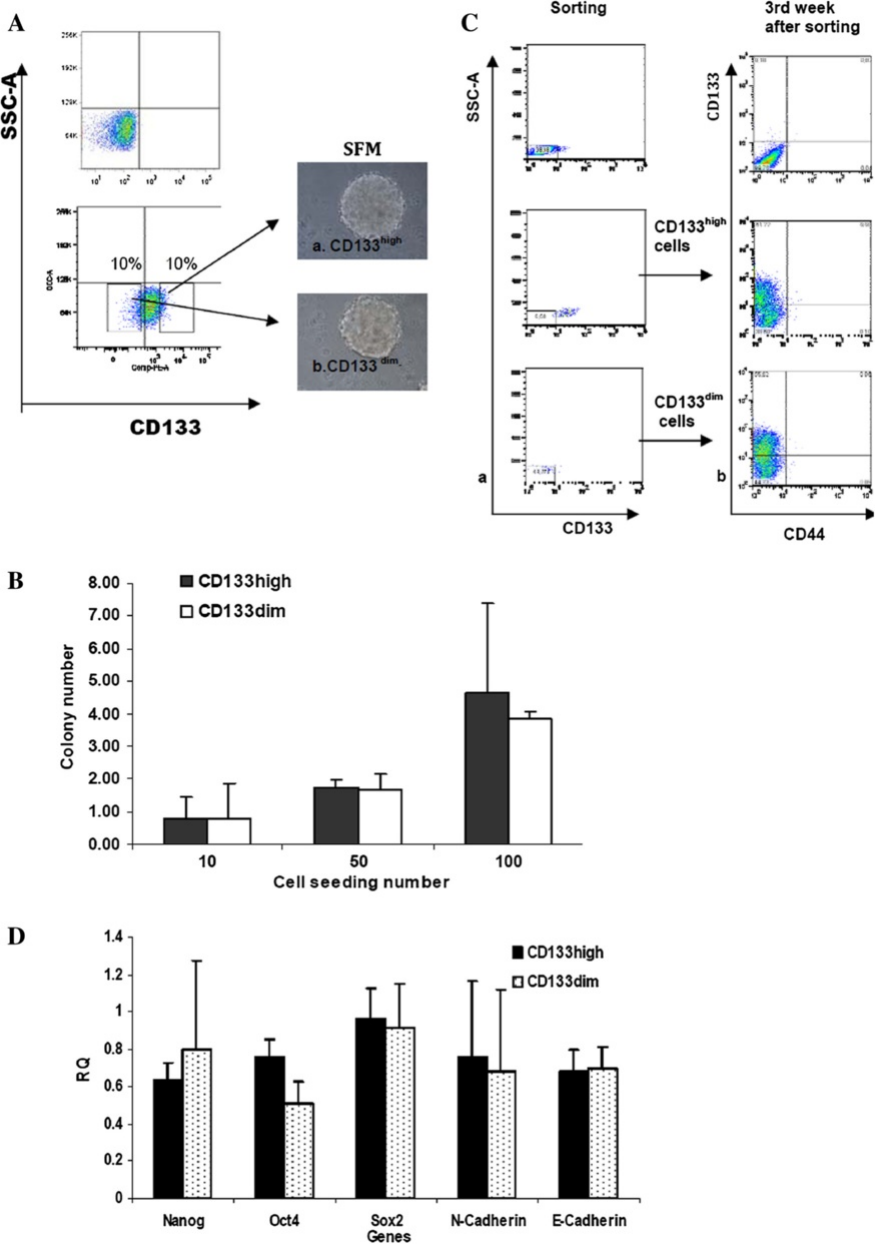
**In vitro analysis of the stemness properties in sorted CD133**^**high **^**and CD133**^**dim **^**LC-42 cells. A**. Cell sphere-forming assay under serum-free condition. 10% highest CD133^+^ cells and the lowest 10% CD133^−^ were isolated via FACS. Spheres were generated from both sorted CD133^high^ and CD133^dim^ LC-42 cell populations within 3 weeks. Representative cell sphere images were acquired at 40× magnification. **B**. Colony-forming ability of different sorted LC-42 cell populations at different cell seeding numbers using soft agar clonogenic assay. The colony numbers of each cell population were compared at different cell seeding numbers. A direct relation between the number of cells seeded and number of colonies formed was observed. The clonogenic ability of different sorted cell populations showed no significant difference. The values of colony numbers of each cell population represented the average of three individual analysis. **C**. Recapitulate phenotype of the original LC-42 cells by post-sorting cell culture. FACS analysis of CD133 just following sorting (column a) and at the third week after sorting under the conventional cell culture condition (column b) showed the expressions of the CD133 in both CD133^high^ and CD133^dim^ cell fractions reached the similar level as its original LC-42 cells at the third week after sorting. **D**. Relative quantitative real-time PCR analysis of the expression of stem cell related genes *Nanog, Oct4* and *Sox2* in sorted CD133^high^ and CD133^dim^ LC-42 cell fractions. Real-time PCR assay revealed similar levels of these genes expression in CD133^high^ and CD133^dim^ cell fractions. The error bar reflected the variation within the triplicates.

When sorted CD133^high^ and CD133^dim^ LC-42 cells were tested in a soft agar colony-forming assay no difference in clononogenic capacity was found between the two cell fractions at each cell seeding density, and a direct relation between the number of cells seeded and number of colonies formed was seen in all experiments (Figure 3B). When the sphere-forming assay was used, both sorted CD133^high^ and CD133^dim^ cells had the capability to form similar numbers of compact cell spheres in serum-free medium (Figure 3A).

### Expression of stemness genes in CD133^high^ and CD133^dim^ LC-42 cells

The mRNA expression levels of *Nanog*, *Oct4* and *Sox2* genes, which are associated with maintenance of stemness, were investigated in the CD133^high^ and CD133^dim^ cell populations by relative quantitative real-time PCR analysis. No significant differences of the gene expression levels were found in the CD133^high^ and CD133^dim^ LC-42 cells (Figure 3D). The epithelial marker *E-Cadherin* and mesenchymal marker *N-Cadherin* were also investigated in the CD133^high^ and CD133^dim^ LC-42 cells, and both cell populations showed same expression levels of these genes.

### Phenotype profile of LC-42 after cell sorting and culturing

FACS sorted CD133^high^ and CD133^dim^ LC-42 cells were maintained in culture under the same culture condition as for the parental unsorted LC-42 cells for three weeks. Thereafter, expression of CD133 was examined. Interestingly, the percentage of the CD133^+^ cells in both sorted CD133^high^ and CD133^dim^ cell populations was identical and reached the same level of CD133 expression as in the parental unsorted LC-42 cells (Figure 3C), indicating that both CD133^high^ and CD133^dim^ cell populations are able to recapitulate the original CD133 heterogeneity found in the parental unsorted LC-42 cells.

### Characterization of SP cells in LC-42

**Figure 4:**
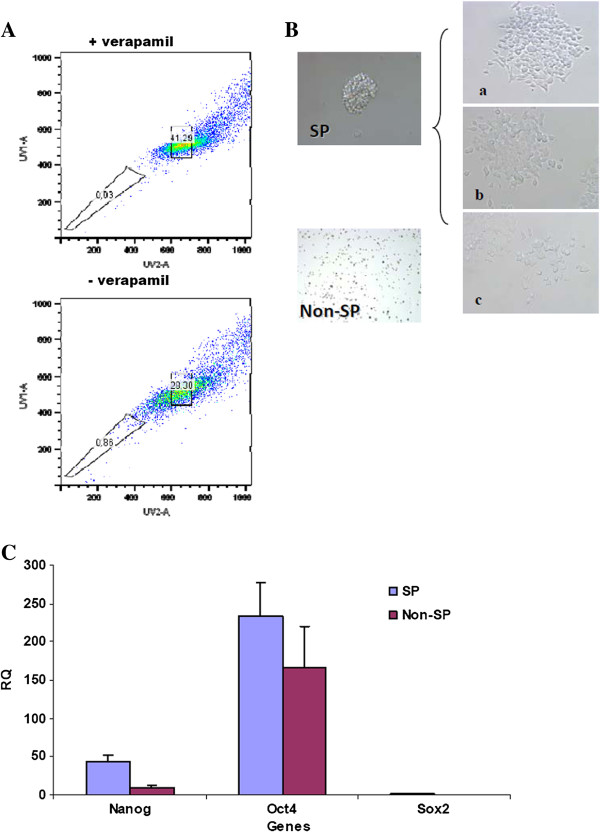
**SP and non-SP fractions in LC-42 lung cancer cells. A**. One representative experiment of the side population in LC-42 cell line as compared to the blocking control with verapamil. **B**. Sorted SP and non-SP cells were cultured in Defined Keratinocyte-SFM medium supplemented with EGF and FGF, only SP cells have capability of forming spheres in serum-free medium (a). These spheres began to attach to the bottom of the wells during the 2nd week under the same culture condition, three distinct patterns of colony morphologies, which were holoclone (a), meroclone (b) and paraclones (c), could be easily identified. Non-SP cells were not able to form cell sphere. Representative colony images were acquired at 40× magnification. **C**. Comparison of the expression pattern of stem cell related genes between SP and non-SP cells by real-time PCR analysis, indicating similar levels of the expression of these genes in SP cells compared to non-SP cells. The error bar reflected the variation within the triplicates. **D**. Colony-forming ability of sorted SP and non-SP LC-42 cells at different cell seeding numbers using soft agar clonogenic assay. The numbers of colonies formed from each cell population were compared at different cell seeding numbers. The clonogenic ability showed significant difference between SP and non-SP cells at lower cell seeding number of 10 cells per tube, showed no significant difference at higher seeding density. The mean value reflected the average of three individual analyses.

SP cells have been suggested to contain tumor cells with stemness properties [23]. When this was investigated in the LC-42, the average SP cell fraction present in the parental LC-42 cells was 0.91±0, 24% (mean±SD, n=8) (Figure 4A). The SP cells and non-SP cells were sorted and additional stemness gene analysis with the relative quantitative real-time PCR method was performed. Compared with the gene expression level in the parental unsorted LC-42 cells, the expression level of *Nanog* gene in the SP cells was much higher than that in the non-SP cells (ratio>5.0). The *Sox2* gene expression level was equally down-regulated in both the SP and non-SP populations (ratio<0.5) (Figure 4C), while there was no significant difference for the *Oct4* gene expression in the individual cell populations studied.

**Figure 5:**
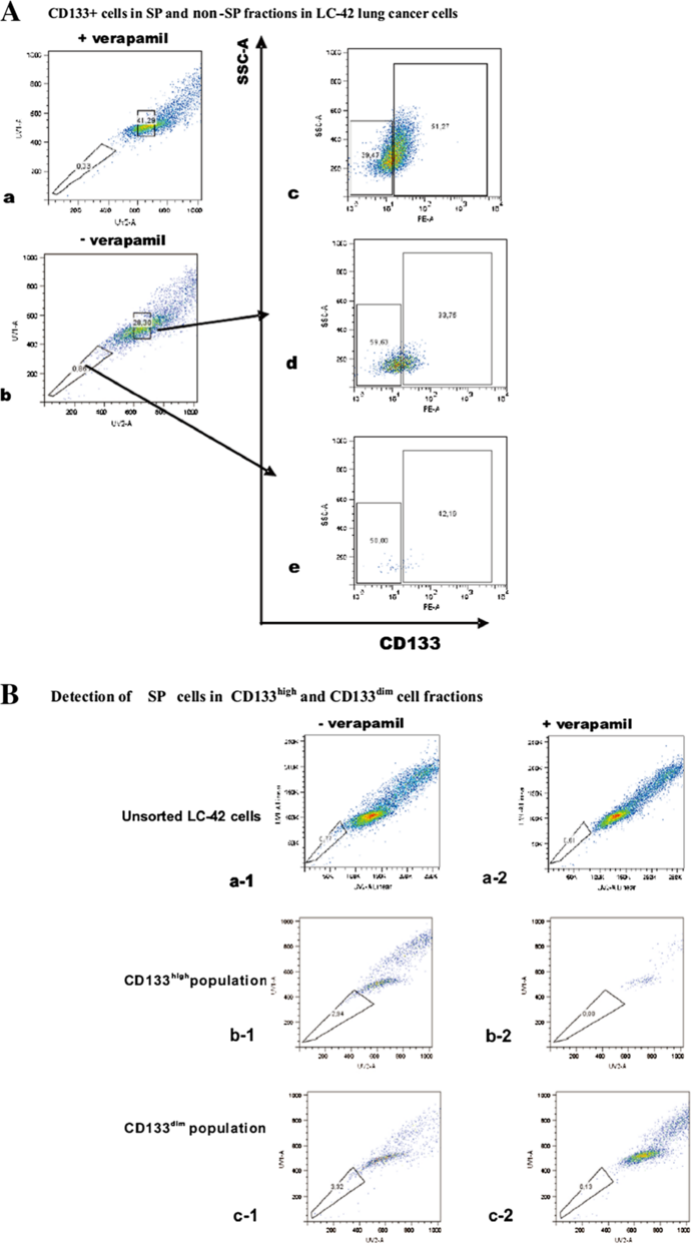
**The correlation between SP cells and CD133 antigen expression in LC-42 cell line. A**. One representative experiment of the CD133 antigen expression in the isolated SP and non-SP cells. The percentage of the CD133^+^ cells in LC-42 cell line is 51.27% (c): percentage of CD133^+^ cells was 33.75% in SP (d) and 42.19% in non-SP (e), demonstrating no significant difference of the CD133 antigen expression between SP and non-SP cells. **B**. One representative experiment of the SP in the isolated CD133^high^ and CD133^dim^ cells. A distinct SP was detected in both isolated CD133^high^ (b-1) and CD133^dim^ (c-1) LC-42 cells, and no significant difference of the SP between CD133^high^ and CD133^dim^ cells. Cells with added verapamil were used as the blocking control (b-2 and c-2).

When CD133 antigen expression was studied in LC-42 SP and non-SP, it was found that the percentages of the CD133^+^ cells in the SP and non-SP fractions were 33.75% and 42.19%, respectively. This confirmed that CD133^+^ cells were not enriched in the SP cells (Figure 5A). Furthermore, distinct SP cells could be obtained in both the CD133^high^ and CD133^dim^ fractions, with about 0.8% and 0.86% respectively, which were similar to the SP percentage discovered in the parental unsorted LC-42 cells (0.91±0.24%) (Figure 5B).

When sphere-forming analysis was performed, it was found that SP cells showed capability to form spheres even when cell seeding density was as low as 6500 cells per well (Figure 4B). The early present cell spheres began to attach to the bottom of the wells during the second and the third week in the serum free medium. From these cultured spheres, three patterns of colony morphology, i.e. haloclone, meroclone and paraclones, were generated (Figure 4B, a, b, c), while non-SP cells were unable to form any cell sphere even with seeding density at 1.0-1.5×10^5^ cells per well. Single suspended cells could be observed in the non-SP after 3 weeks culture (Figure 4B), but additional analyses showed that these cells were apoptotic.

### In vivo tumorigenesis assay

**Figure 6:**
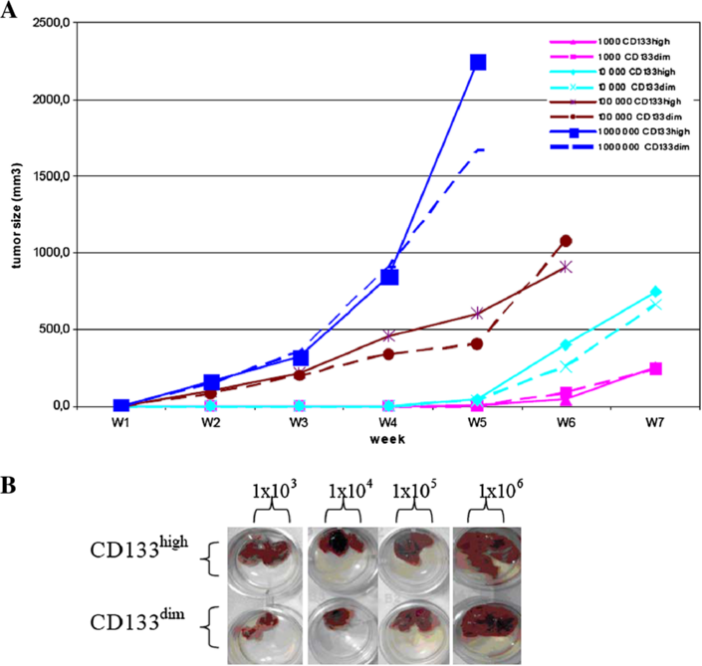
**In vivo tumorigenicity assay of FACS sorted CD133**^**high **^**and CD133**^**dim **^**LC-42 cells in NOD/SCID mice. A**. Comparison of xenograft formation in NOD/SCID mice with different injected numbers of CD133^high^ and CD133^dim^ cells. Injected cell dose was found to be related with the time of the bulk tumor appearance and tumour size. When 1000 cells were injected, both CD133^high^ and CD133^dim^ LC-42 cells formed tumors at the 5th week, while only 2 weeks was required when 1×10^6^ cells were injected. **B**. Representative xenografts from the CD133^high^ and CD133^dim^ LC-42 cells derived from the different injected cell doses. No significant difference of growth appeared between CD133^high^ and CD133^dim^ cells.

Both CD133^high^ and CD133^dim^ sorted LC-42 cells were 100% tumorigenic at every cell dose even when 1000 cells were inoculated in the animals. As expected from the results of in vitro experiments, the time to tumor formation was related to the number of cells xeno-transplanted. Thus, when transplanting 1000 cells of sorted CD133^high^ and CD133^dim^ LC-42 cells, six weeks was needed to get tumours formed, while only 2 weeks was required when 1×10^6^ cells were injected (Table 3). Altogether our in vivo results showed no difference in tumour growth between the CD133^high^ cells and CD133^dim^ LC-42 cells (Figure 6A, B), indicating that the CD133 antigen expressing cells have no growth *in vivo* advantage when compared to the CD133^dim^ LC-42 cells. Furthermore, both CD133^high^ and CD133^dim^ cells were able to generate tumors in the second transplantation and no differences were seen in the CD133^high^ and CD133^dim^ cell populations with regard to both tumor size and tumor characterization.

**Table 3:** **In vivo tumorigenicity assay of FACS sorted CD133**^**high **^**and CD133**^**dim **^**LC-42 cells at different injected cell doses in NOD/SCID mice**

**Group**	**Cell type**	**Number of the cells injected**	**Number of the mice with tumor/ number of the mice injected**	**Latency**
**No.**	**injected**			
**1**	CD133^high^	1×10^3^	3/3	6W
**2**	CD133^dim^	1×10^3^	3/3	6W
**3**	CD133^high^	1×10^4^	3/3	5W
**4**	CD133^dim^	1×10^4^	3/3	5W
**5**	CD133^high^	1×10^5^	5/5	3W
**6**	CD133^dim^	1×10^5^	5/5	3W
**7**	CD133^high^	1×10^6^	3/3	2W
**8**	CD133^dim^	1×10^6^	3/3	2W
**1**	Unsorted cells	1×10^5^	3/3	3W
**2**	Unsorted cells	2×10^5^	3/3	3W
**3**	Unsorted cells	2×10^6^	3/3	2W
**4**	Unsorted cells	5×10^6^	6/6	2W

Comparison of xenograft formation in NOD/SCID mice with different numbers of injected CD133^high^ and CD133^dim^ cells. Both FACS isolated CD133^high^ and CD133^dim^ LC-42 cells were 100% tumorigenic in NOD/SCID mice at different injected cell dose even only 1×10^3^ cells were injected. Unsorted LC-42 cells were established as control groups.

The xenograft samples were embedded into paraffin blocks and sections from these blocks were prepared for additional IHC studies. A clear CD133 expression could be immunohistochemically disclosed in all the xenografts, including the xenografts from the sorted CD133^high^ cells, CD133^dim^ cells and unsorted parental LC-42 cells, with approximately the same intensity. Strong expressions of OCT4, SOX2, SSEA1 and SSEA4 were seen in all the xenograft samples (Figure 2A) (Table 2). In addition, the pan-epithelial marker Ber-EP4 was strongly positive in all the xenograft samples, verifying their epithelial cell origin.

## Discussion

The cell surface marker CD133 has been associated with tumor stem cells in lung cancer [20]. Following phenotyping of 4 human lung cancer cell lines, we found that only the LC-42 cells expressed high levels of CD133. When the LC-42 cell line was tested further both *in vitro* or *in vivo*, we found that CD133 antigen expression in LC-42 cells was not identifying cells with CSC/TIC properties.

Many investigators have attempted to identify and isolate CSC/TIC using different surface markers. The CD133, a surface glycoprotein linked to organ-specific stem cells, has frequently been used to identify CSC/TIC from fresh human tumour cells in brain [7], pancreatic [11] and colon [9,10]. Purified CD133^+^ cells from these types of cancers show self-renewal capacity and have ability to form tumours in transplanted immunodeficiency mice. The CD133 expressing cells from fresh tumor samples were also found to have cancer-initiating properties in both small cell and non-small cell lung cancers. Furthermore, as few as 1000 CD133^+^ cells were capable of inducing tumors in immunedeficient SCID or nude mice [20]. CD133 antigen expression at various levels was detected in various human cancer cell lines [24,25]. In the liver cancer cell line Huh-7 and colon cancer cell line Caco-2, the CD133 was expressed in 50% [24] and 70% [26] of the tumor cells respectively. When CD133^+^ cells in some of these cell lines was further investigated, it was found that CD133 expressing tumor cells possessed high capacity for tumorigenicity. Since our lung cancer cell line LC-42 had similar CD133 antigen expression as those cell lines, we decided to further characterize the LC-42 human lung cancer cells.

In the present study, seven surface markers were used to characterize the cell lines. Interestingly, we found a correlation between the antigens CD133 and CD44 expression on the individual lung cancer cell lines tested. While the LC-42 had high expression of CD133, no or weak expression of CD44 could be detected on the cell line. In contrast, the cell lines HTB-182, EKVS and SELS all show high expression of CD44 but weak or no expression of CD133. These findings were also confirmed by immunocytochemistry using the CD133 antigen clone AC133.

The differences observed in CD133 and CD44 expression cannot be explained by which type of primary lung cancer the cell line originated from, since both the HTB-182 and LC-42 were derived from squamous cell carcinoma while the EKVX and SELS cells were derived from adenocarcinoma. Recently, it has been reported that CD44 is a marker in lung cancer cell lines that define a subpopulation of tumor initiating cells [27]. In our study, no expression of CD133 was found in the cell lines HTB-182, EKVS and SELS while all these cell lines were highly positive for CD44 expression.

To investigate the role of CD133 in the LC-42 lung cancer cells we characterized FACS sorted CD133^high^ and CD133^dim^ cells both *in vitro* and *in vivo.* Transcription factors OCT4, SSEA4, SSEA1 and SOX2 are related to stemness properties [28]. Therefore, both IHC and real-time PCR of LC-42 cells were performed to explore the expression profiling of transcription factors. IHC studies showed that SSEA1 protein expression was strongly positive in the LC-42 cell line, LC-42 xenografts, the CD133^high^ and the CD133^dim^ xenografts. A weaker expression of OCT4, SSEA4 and SOX2 was seen by IHC, but no difference was found in the various subtypes of LC-42 cells (Figure 2A), which is in line with the real-time PCR analysis of *Nanog, Oct4 and Sox2.* As a physiological process that occurs during various stages of embryogenesis, Epithelial-mesenchymal transition (EMT) program has been reported also to play a crucial role during cancer invasive growth and metastases [29]. EMT may facilitate the cancer cells with the mesenchymal traits needed for dissemination as well as self-renewal property for initiation of secondary tumors and increasing evidence shows a direct link between EMT and the CSCs [30]. In our study there were no differences in the expression of *N-Cadherin* and *E-Caderin* in the sorted CD133^high^ and CD133^dim^ LC-42 cells, suggesting no EMT shift in these two different cell populations.

*In vitro* colony and sphere formation assays were performed to test whether there was any difference in clonogenic growth among sorted CD133^high^ and CD133^dim^ cells. Our results (Figure 3) show that both the CD133^high^ and CD133^dim^ LC-42 cells exhibit equal growth potential in vitro when examined with the 2D and 3D colonogenic methods. If CD133^high^ cells are the only population that contains tumor initiating cells it would be expected that CD133^dim^ cells do not give rise to CD133^high^ cells following further culture. However, when sorted CD133^high^ and CD133^dim^ cells were maintained in culture for 3 weeks, it was found that both cell fractions were able to return to the CD133 expression level of the parental LC-42 cells, indicating that CD133^high^ cells are not defining cells with cancer stem cell properties.

It has been shown that cells with stem cell properties from different types of normal tissues have a unique ability to efflux Hoechst 33342 dye by an ATP-binding cassette (ABC) transporter [31]. These cell populations can be be defined by flow as a side population (SP). Increasing evidence indicates that cells in the SP fraction in different human primary cancers as well as in cell lines contain tumour-initiating cells [23,32-35]. This has also been found in primary lung cancer [36]. The lung cancer cell line LC-42 also displayed persistent SP cells representing around 1% (0.91±0.24%) of the entire LC-42 cell population. Following culturing in defined serum-free medium, SP cells had significant higher cell sphere-forming potential compared with non-SP cells. Furthermore, the transcription factor *Nanog* was highly expressed in the SP fraction, suggesting that SP cells in the LC-42 cell line may contain cells with greater stemness properties. When we further studied the relation between SP cells and CD133^+^ expression in the LC-42 cells, no difference was found in the SP and non-SP cells for CD133 expression, and in line with this no difference in the colony formation assay between the CD133^high^ SP and CD133^dim^ SP was seen (data not shown).

The gold standard used for identifying CSC is that the candidate cell population must be able to initiate serially xenograft tumors recapitulating the original tumor. In our study, all animals showed tumor growth irrespectively when unsorted, sorted CD133^high^ and CD133^dim^ cells were injected and as few as 10^3^ CD133^high^ and CD133^dim^ cells were sufficient to form tumors in all animals. The CD133^high^ and CD133^dim^ cells from the xenografts could be serially transplanted without losing their ability to form tumors. IHC staining of the xenografts derived from the sorted CD133^high^ and CD133^dim^ cells showed no difference in stemness related gene expression, in comparison to the parental LC-42 cells. In addition, both the CD133^high^ and CD133^dim^ cells formed tumors, which were similar to the tumors derived from the parental LC-42 cells. Furthermore, it was also observed that the tumors derived from the CD133^dim^ LC-42 cells were equally positive for the CD133 expression as in the tumors derived from the CD133^high^ cells and their parental LC-42 cells, further suggesting that both subpopulations contain similar numbers of cancer-initiating cells.

Recently Shmelkov *et al.* challenged the view that CD133 is a marker of CSC/TIC in colon cancer. In their study it was found that both CD133^+^ and CD133^-^ antigen expressing cells from metastatic colon cancers isolated from liver were equally capable of initiating tumors in mice [22]. Our results on the lung cancer cell line LC-42 and CD133 antigen expression are in line with Shmelkov’s observation. In contrast to the other lung cancer cell lines studied, the LC-42 was also established from a lymph node metastasis 3 months after the patient had been treated with radiotherapy combined with chemotherapy (Taxans).

In summary, we conclude that in the present study, CD133 expression in the human lung cancer cell line LC-42 does not determine in vitro as well as in vivo a cell population with tumor initiating properties. Therefore, other surface markers on lung cancer cells should be explored in order to identify subpopulations of cells with stemness properties.

## Materials and methods

### Human lung cancer cell lines and growth conditions

Four human lung cancer cell lines HTB-182, LC-42, EKVX and SELS were used in the present study. Only the HTB-182 cell line, which was established from a sample of a lung mass taken from a patient with primary lung squamous carcinoma, was purchased from ATCC (American Type Culture Collection). All the other three cell lines were established at the Department of Tumour Biology in our hospital. The EKVX cell line was developed from a xenograft from a lung mass taken from a patient with primary lung adenocarcinoma. Two cell lines were established from lymph node metastasis of lung cancer patients: The LC-42 cell line originated from lung squamous carcinoma and the SELS cell line from a lung adenocarcinoma. All cell lines were cultured in standard coated flasks (Nunclon™Δ Surface; NUNC, Denmark) at 37°C with 5% CO_2,_ using RPMI-1640 medium (GIBCO, USA) supplemented with 10% Fetal Bovine Serum ([FBS]; Sera-lab; Sussex, UK) and 2mM Glutamine.

### Phenotyping of lung cancer cell lines by flow cytometry

Prior to harvesting, the cell lines were grown until a 75% confluence. Following detachment of cells with 0.25% Trypin/EDTA (Lanzo, Belgium) and washing with Dulbecco's phosphate-buffered saline (DPBS, Invitrogen,USA), the cells were enumerated and transferred into 12×75 mm polystyrene round-bottom test tubes (BD Falcon, USA) at a cell concentration of 1×10^6^ cells/ml. A staining buffer with gamma globulin at concentration of 1mg/ml (Gammagard, Baxter, UK) was added in order to block FcR. Titrated amounts of phycoerythrin (PE) conjugated mouse anti-human monoclonal antibodies Ber-EP4, CD34, CD45, CD31, VEGFR2, CD117 and CXCR4, or fluorescein isothiocyanate (FITC)-labelled CD44 (all from BD Pharmingen, USA) and PE-labelled CD133 (clone2/AC141 and clone1/AC133, Miltenyi Biotech, Germany) were added to the test tubes and incubated for 15–20 minutes on ice avoiding light exposure. Excess antibody was removed by washing the cells twice with DPBS. Five microliters (μl) of 7-AAD (BD Bioscience, USA) was added into each sample to exclude the dead cells before analysis by flow cytometry. Cells stained with isotype-matched monoclonal antibodies (BD Pharmingen, USA) served as negative controls. The cells were analysed on a FACSCalibur™ and the CELL Quest software (BD Bioscience, USA).

### Immunohistochemistry

Cell blocks were prepared from each of the cell lines. Following culturing of the cell lines into 80% confluence, the cells were washed with DPBS, detached by 0.25% Tripsin/EDTA (Sigma), harvested and centrifuged at 300g for 5 minutes. The supernatant was carefully removed before 3 drops of plasma and 2 drops of thrombin were added and mixed by tube rotation. After the mixture was coagulated, 4% buffered formalin was added. The coagulated mass was then placed in linen paper for further conventional paraffin block preparation.

For immunocytochemistry (IHC), four-micrometer thick sections from the above prepared blocks were dewaxed and rehydrated in graded ethanol. To unmask epitopes, sections were then microwaved in different optimised buffers for different antibodies. Tris/EDTA buffer at pH 9.0 was used for CD133 antibody; low pH 10 mM citrate buffer (pH = 6.0) was used for antibodies SOX2, SSEA1, SSEA4, OCT4 and Ber-EP4. To inhibit endogenous peroxidase, the sections were incubated with 3% hydrogen peroxide (Dako Cytomation) at room temperature for 5 minutes. Thereafter the primary antibodies were added onto the sections. Sections with mouse anti-human CD133 (clone AC133/1 Miltenyi, 1:40 dilution), mouse anti-human SOX2, SSEA1, SSEA4 and Ber-EP4 (R&D, 1:200, 1:100,1:100 and DAKO 1:200 dilution, respectively), and goat anti-human OCT4 (R&D, 1:100 dilution) were incubated overnight at 4°C. The sections were then incubated with corresponding secondary antibodies for 30 minutes at room temperature and stained with 3,3'-diaminobenzidine tetrahydrochloride (Dako EnVision™ + System Peroxidase (DAB), K4007, DakoCytomation, CA, USA) before counterstained with hematoxylin, dehydrated and mounted in Diatex. All sections were rinsed thoroughly with TBS-Tween wash buffer (Dako Cytomation) after each incubation step. Sections from a human seminoma specimen were used as positive controls for antibodies OCT4, SSEA1 and SSEA4; Sections from a human gastrocarcinoma specimen were used as the positive control for antibody SOX2; Sections from colon cancer cell line CaCo-2 were used as the positive control for CD133 antibody; Sections from human breast cancer specimen were used as the positive control for antibody Ber-EP4. Negative controls included substitution of the antibodies with corresponding normal immunoglobulins of the same concentration. All controls were satisfied before experimental applications of the antibodies. Positivity was scored as follows: low positivity (+) for 0–30% positive cells; moderate positivity (++) for 30–70% positive cells; and strong positivity (+++) for over 70% positive cells; no immunoreactivity was scored as negative.

### FACS cell sorting and side population assessment

Directly conjugated Phycoerythrin (PE)-labeled CD133 (clone2/AC141, Miltenyi Biotech) was added in saturated amount and incubated for 30 minutes on ice avoiding light exposure. The cells were thereafter washed twice in cold RPMI-1640 medium. The IgG1-PE was used as isotype control. Before sorting, the cells were filtered through a 40 μm cell-strainer cap (BD Falcon, USA) and then kept on ice. The top 10% CD133^+^ cells and the lowest 10% CD133^-^ cells (defined as CD133^high^ cells and CD133^dim^ cells, respectively, in the description) were gated and sorted. Side population (SP) assay was performed by Hoechst 33342 staining (Sigma-Aldrich, USA). Briefly, cells were resuspended at 10^6^ cells per milliliter in RPMI1640 medium containing 2% FCS. The cells were then incubated with 2.5 μg/ml Hoechst 33342 at 37°C for 90 minutes, which was found to be the optimal time point in our studies (data not shown). To set the gate for SP cells, Verapamil (Boehringer-Mannheim) was included at 50 μM together with the Hoechst dye in control sample. In experiments where both SP and CD133 antigen profiling were performed, CD133 antibody was added after the Hoechst staining prior to flow cytomety analysis. Cell sorting was performed on a FACSDiva cell sorter (Flow Vantage machine, Becton Dickinson). The viabilities of each subpopulation after sorting were checked using Trypan Blue (Sigma Chemical Co., St. Louis, MO).

### Soft agar colony-forming assay

The colony-forming capability of the different subpopulations derived from the LC-42 cells following sorting by SP and CD133 antigen was evaluated in a soft agar assay as described previously [37]. Briefly, soft agar cultures were set up in triplicates in 10ml tubes by adding 0.2 ml of August rat blood diluted 1:8, 0.2 ml of appropriately diluted tumor cell suspensions, and 0.6 ml of 0.5% agar (DIFCO Laboratories, Detroit, MI). After plating different numbers of the sorted subpopulations of LC-42 cells in triplicate, the tubes were incubated at 37°C in a low-oxygen culture incubator. Following 3 weeks of incubation, colonies containing more than 50 cells were recorded as positive using an inverted microscope, and the number of colonies were counted.

### Sphere-forming analysis in serum-free medium

LC-42 cells were sorted into CD133^high^ and CD133^dim^ subpopulations and SP and non-SP subpopulations. The LC-42 CD133^high^ and CD133^dim^ cells were seeded at a cell density of 300 cells/well in 6-well plates. SP and non-SP cells were seeded at a density of 6500 cells/well and 1.0×10^5^ cells/well in 6-well plates, respectively. Culture condition consisted of Defined Keratinocyte-SFM medium (Invitrogen, USA) complemented with EGF 20 ng/ml and FGF 10 ng/ml (Peprotech, USA). Sphere morphology was observed and the numbers of spheres were then counted.

### RNA extraction and real-time PCR analysis

Sorted cells were collected and total RNA was extracted from the sorted CD133^high^ and CD133^dim^ subpopulations respectively by TRIzol reagent (Invitrogen USA) according to the manufacturer's instructions, and subsequently reverse transcribed using the High Capacity RNA-to-cDNA Kit (Applied Biosystems, USA). Fifty ng of total RNA was used as template. Incubate conditions were 16°C for 30 min, followed by 42°C 30 min, 85°C 5 min. Relative quantitative real-time analysis was performed by the ABI 7500HT Real-Time PCR System (Applied Biosystems, USA). Thermal cycling conditions were 95°C for 10 min, followed by 40 cycles of 95°C 15s, 60°C 1min. All samples, including the no template controls, were assayed in duplicate. PCR reaction without template was served as negative control. The expression levels were determined by TaqMan Gene Expression Assays (Applied Biosystems, USA) for the genes *Nanog* (assay ID 8S02387400_gl), *Oct4* (assay ID Hs03005111_g1), *Sox2* (assay ID Hs01053049_sl), *E-Cadherin* (assay ID Hs01023895_m1) and the mesenchymal marker *N-Cadherin* (assay ID Hs00169953_m1), The gene *phosphoglycerate kinase 1* (*PGK1,* assay ID Hs99999906_m1) was used as endogenous control for normalization of gene expression, and unsorted LC-42 cells were used for calibration. Data were analyzed by the 7500 System Sequence Detection Software, The Comparative C_T_ method, which was ddCT, was used to calculate relative transcripts levels.

### Mouse xenograft studies

All animal experiments were approved by and performed according to the guidelines set by the Animal Research Ethics Board at University of Oslo. Four to five weeks old non-obese diabetic/severe combined immunodeficiency (NOD/SCID) male mice were used in all experiments. The research was done in collaboration with the animal facility in the Norwegian National Hospital. Different numbers of sorted CD133^high^ and CD133^dim^ LC-42 cells were mixed with Matrigel (BD Biosciences, San Diego, CA) at a ratio of 1:1 and injected subcutaneously into the right flank of NOD/SCID mice at a maximum volume of 150 ul. Unsorted LC-42 cells were injected as positive control. The animals were monitored twice a week and tumor size was measured with callipers. Mice were sacrificed when the size of the tumor reached a diameter of 1.5 cm. Tumor volumes were determined using the formula: width^2^×length/2. The paraffin blocks were prepared from the xenografts. The expression of CD133 in the CD133^high^ and CD133^dim^ xenografts were immunohistochemically examined in comparison to their original cell line LC42. The expression of Ber-EP4, stemness transcription factors OCT4, SSEA1, SSEA4, and SOX2 were also studied in the xenografts immunohistochemically.

## Competing interests

The authors have declared that no competing interests exist. Author contributed equally to this paper.

## Authors’ contributions

PW GK: conceived and designed the experiments; PW MW: performed the experiments; PW ZS GK: analyzed the data; PW ZS HKH GK GG: contributed reagents/materials/analysis tools; PW: wrote the manuscript; ØF GK GG: revised the article critically for important content. All authors read and approved the final manuscript.

## Supplementary Material

Additional file 1: Table S1 IHC analysis of Ber-EP4, OCT4, SOX2, SSEA1 and SSEA4 in the lung cancer cell lines. Click here for file
