# Supplementary material for: In vitro and in vivo properties of CD133 expressing cells from human lung cancer cell lines
Source: Exp Hematol Oncol. 2013 Jun 6;2:16. doi: 10.1186/2162-3619-2-16 (PMC3734134; doi:10.1186/2162-3619-2-16)
Supplement: Additional file 1: Table S1 — IHC analysis of Ber-EP4, OCT4, SOX2, SSEA1 and SSEA4 in the lung cancer cell lines. [file 2162-3619-2-16-S1.doc]

**Table S1: IHC analysis of Ber-EP4, OCT4, SOX2, SSEA1 and SSEA4 in the lung cancer cell lines.**

| **Cell line**  **Marker** | **LC-42** | **HTB-182** | **EKVX** | **SELS** |
| --- | --- | --- | --- | --- |
| **Ber-EP4** | **+** | **+** | **+** | **+** |
| **OCT4** | **+** | **-** | **+** | **+** |
| **SOX2** | **+** | **+** | **Single cell +** | **-** |
| **SSEA1** | **+** | **Single cell +** | **-** | **+** |
| **SSEA4** | **+** | **+** | **+** | **+** |

**The summary of the IHC analysis of the pan-epithelia marker Ber-EP4 and stem cell related transcription factors** **OCT4, SOX2, SSEA1 and SSEA4 in the 4 lung cancer cell lines: LC-42, HTB-182, EKVX and SELS.**
